# Hotspots of N2O accumulation in the soil profile of alternate wetting and drying paddy fields

**DOI:** 10.3389/fpls.2026.1756990

**Published:** 2026-02-05

**Authors:** Guangyan Liu, Xuda Chen, Taotao Chen, Daocai Chi, Hongtao Zou

**Affiliations:** 1 Postdoctoral Station of Agricultural Resources and Environment, Land and Environment College, Shenyang Agricultural University, Shenyang, China; 2 College of Land and Environment, Shenyang Agricultural University, Shenyang, China; 3 College of Water Conservancy, Shenyang Agricultural University, Shenyang, China

**Keywords:** alternate wetting and drying irrigation, hotspot, nitrous oxide, paddy fields, soil profile

## Abstract

**Introduction:**

Alternate wetting and drying irrigation (I_AWD_) is a promising practice for water conservation and climate mitigation, yet it inadvertently stimulates substantial nitrous oxide (N_2_O) emissions. While previous research has largely focused on surface N_2_O fluxes, the processes governing N_2_O accumulation and emission across the soil profile–surface continuum remain poorly understood.

**Methods:**

Here, we present a comprehensive dataset from a lysimeter study on paddy fields under I_AWD_ and continuously flooded irrigation (I_CF_), integrating measurements of soil N_2_O concentrations (0–50 cm depth, at 10-cm intervals) and concurrent surface fluxes.

**Results:**

The results showed that N_2_O predominantly accumulated in 0–20 cm soilprofiles during the tiller fertilizer period (TF) and panicle fertilizer period (PF) regardless of the irrigation regimes. Compared to I_CF_, I_AWD_ significantly increased the N_2_O concentrations in 0–30 cm soil profiles by 19.6–49.3% and 60.0–79.0% during the TF and PF, respectively. Partial least-squares path model further identified the 10–20 cm layer as the dominant hotspot, exerting the strongest direct control on surface N_2_O emissions.

**Discussion:**

Altogether, 0–20 cm soil profiles are the hotspots for N_2_O accumulation in I_AWD_ paddy fields, and the N_2_O accumulated in 10-20 cm soil profile dominates the N_2_O emissions. These findings contribute to the adoption of straightforward and targeted N_2_O mitigation strategies in I_AWD_ paddy fields.

## 1 Introduction

Rice, a staple crop vital for global food security, faces a pressing challenge at the water-climate nexus (Jiang et al., 2017). Conventional paddy irrigation accounts for nearly 40% of agricultural water withdrawals globally—a demand intensified by climate change—while growing water scarcity increasingly threatens production sustainability (Yao et al., 2012; Konapala et al., 2020; Jägermeyr et al., 2021; Zhou et al., 2021). In response, alternate wetting and drying irrigation (I_AWD_) has emerged as a promising strategy, demonstrating significant potential to reduce water use and mitigate methane emissions without compromising yield (Ishfaq et al., 2020; Sha et al., 2022; Zhao et al., 2023; Xing et al., 2025). This practice periodically introduces aerobic phases into traditionally anaerobic paddy soils by cyclically draining and re-flooding fields (Chen et al., 2025). However, this very mechanism, which suppresses methane production, concurrently promotes substantial nitrous oxide (N_2_O) emissions during the controlled drying periods, presenting a critical trade-off between water conservation and climate impact (Liu et al., 2022).

The adverse effect of I_AWD_ on N_2_O emissions has been extensively documented in the field, regional, and global scales (Jiang et al., 2019; Zhao et al., 2024; Xing et al., 2025). For instance, a global meta-analysis of 636 published observations worldwide found that non-continuous flooding practices increased N_2_O emissions by 92% (Bo et al., 2022). The mechanisms underlying these substantial bursts of N_2_O are likely complex biological processes. On the one hand, the higher N_2_O emissions under I_AWD_ are primarily caused by the increased substrate of nitrification-denitrification (Verhoeven et al., 2018). Specifically, I_AWD_ disrupts soil aggregates and ruptures microbial cells, resulting in the release of nitrogen-containing compounds as substrates of nitrification and denitrification (Kraus et al., 2022; Liu et al., 2022). On the other hand, the frequent alternation of the oxidative and reductive environment contributes to substrate decomposition and higher O_2_ concentration, thereby promoting N_2_O production (Kravchenko et al., 2017; McCoy et al., 2023). Overall, previous studies have confirmed that I_AWD_ leads to a substantial increase in N_2_O production and subsequent emissions mainly through microbial- and soil environment-mediated mechanisms. However, the mechanisms mentioned above fail to directly consider the N_2_O accumulation in soil profiles, which leaves us with an insufficient understanding of the linkage between N_2_O production and emission.

It should be noted that the study of soil profile N_2_O accumulation should take priority over N_2_O surface emission, which could lower the barrier to adopting effective mitigation technologies. Indeed, several studies have explored the accumulation of N_2_O in soil profiles and build the relationship between soil profile N_2_O accumulation and surface emissions (Yin et al., 2019; Gao et al., 2014). For example, in drip-fertigated cotton fields, Li et al. (2021) found peak N_2_O concentrations at 30 cm depth, while surface emissions were primarily linked to the 0–15 cm layer. They suggested that using efficient N fertilizers and appropriate application methods to reduce topsoil nitrogen accumulation can effectively mitigate N_2_O emissions. Taken together, identifying soil depths that are responsible for N_2_O accumulation processes can provide direct guidance in the practical placement of fertilizers. Meanwhile, it is of utmost importance to bridge the soil profile N_2_O and surface N_2_O emissions to manage agricultural strategy for building climate-smart and resource-efficient agroecosystems. However, the existing studies on soil profile N_2_O accumulation are in dryland agroecosystems, and few are in paddy fields, especially under I_AWD_. Given the widespread implementation of I_AWD_ in global rice cultivation and the significant N_2_O source induced by I_AWD_ (Bo et al., 2022; Chen et al., 2022), research should encompass the soil profile-surface continuum to explore the N_2_O accumulation and emission characters. Such efforts would lower the barrier to adopting straightforward and targeted mitigation strategies in I_AWD_ paddy fields.

To this end, based on a long-term water management experiment (i.e., commenced in 2014 including I_CF_ and I_AWD_), we conducted 2-year *in-situ* field measurements to monitor N_2_O concentration in soil profiles (0–50 cm soil depth, 10 cm per layer) and soil surface N_2_O flux in I_CF_ and I_AWD_ paddy fields from northeast China. The objectives of this study were to (1) identify the hotspots for soil profile N_2_O accumulation in I_AWD_ paddy fields, (2) build the connection between soil profile N_2_O accumulation and emission in I_AWD_ paddy fields, and (3) quantify the contributions of soil profile N_2_O accumulation to N_2_O emissions in I_AWD_ paddy fields. Our findings will provide the information for understanding the N_2_O accumulation and emission along the soil profile-surface continuum, which could help researchers and policy-makers develop and adopt straightforward and targeted N_2_O mitigation strategies in I_AWD_ paddy fields.

## 2 Materials and methods

### 2.1 Site description

**Figure 1:**
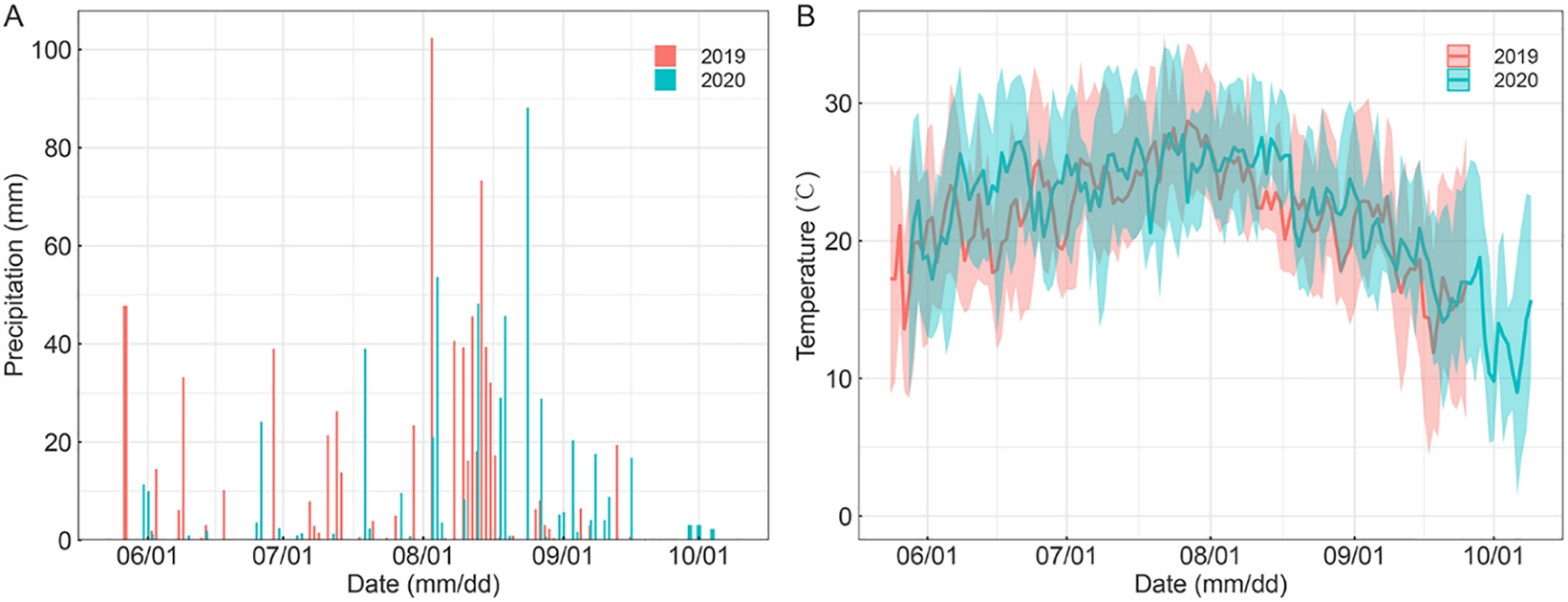
Daily **(A)** precipitation and **(B)** air temperature during two rice-growing seasons in 2019 and 2020.

A two-year field experiment was conducted during the rice growing seasons (May–October) of 2019 and 2020 at the Liaoning Irrigation Experiment Center Station (42°08′59″N, 120°30′44″E; 47 m altitude) in Shenyang, Liaoning Province. The site experiences a temperate continental monsoon climate with a mean annual temperature of 7.5°C and annual precipitation of 673 mm. Local meteorological data during the study period were recorded by an on-site automatic weather station (**Figure 1**). The soil is classified as clay loam, with the topsoil (0–15 cm) containing organic matter 22.3 g kg^−1^, total nitrogen 0.78 g kg^−1^, alkali-hydrolyzable nitrogen 75.4 mg kg^−1^, Olsen-P 18.4 mg kg^−1^, exchangeable potassium 81.3 mg kg^−1^, pH 7.40, and bulk density 1.50 g cm^−3^. A mid-late season rice cultivar (Oryza sativa L. cv. Shennong 9765), characterized by high yield, superior grain quality, and strong disease resistance, was used in this study.

### 2.2 Experimental design

The experiment followed a randomized complete block design with two irrigation treatments—continuous flooded irrigation (I_CF_) and alternate wetting and drying irrigation (I_AWD_)—each replicated three times. Six concrete lysimeters (2.5 × 2 × 1.5 m) were used, each equipped with an individual water gauge and meter to precisely control irrigation. Waterproof sealing prevented lateral water and nutrient exchange, and a movable rainout shelter excluded natural precipitation. In I_CF_ plots, a 1–5 cm flooding depth was maintained from transplanting until two weeks before harvest. In I_AWD_ plots, standing water (1–3 cm) was maintained for the first two weeks after transplanting, after which fields were allowed to dry until the soil water potential at 0–15 cm depth reached –15 kPa, as monitored by installed tensiometers. Floodwater was then reapplied, and this cycle was repeated until two weeks pre-harvest. Soil moisture is monitored by recording water table and tensiometers at 8 a.m. and 2 p.m. every day to strictly control water in accordance with the irrigation regime. The experimental setup and agronomic management were consistent with a long-term study initiated in 2014 assessing I_AWD_ effects on rice yield, water productivity, and grain quality. This long-term alternate wetting and drying irrigation experiment initially primarily explored the effects of alternate wetting and drying irrigation on rice growth, water use efficiency, and rice yield. Subsequently, it investigated the impacts on rice photosynthetic characteristics, dry matter accumulation, and rice quality. Recently, it has mainly focused on its environmental effects, including leaching losses, greenhouse gas emissions and the impacts on the cycling of carbon, nitrogen and other nutrients (Zheng et al., 2018a, 2018b; Liu et al., 2022).

Seedings were manually transplanted on 24 May 2019 and 28 May 2020 to each completely cleaned, plowed, and flatted plot. Inter and inter-row spacing of seedings were 16 cm and 30 cm with four seedings per hill, respectively. A total of 210 kg ha^−1^ urea (46% N) was applied as N fertilizer (43% in the basal fertilizer period, 43% in the tiller fertilizer period, and 14% in the panicle fertilizer period), a total of 60 kg ha^−1^ P_2_O_5_ (12%) was applied as P fertilizer (100% in the basal fertilizer period) and a total of 37.5 kg ha^−1^ K_2_O (50%) was applied as K fertilizer (50% in the basal fertilizer period, 50% in the tiller fertilizer period). Diseases and insects were controlled with chemicals, and weeds were manually cleaned by farmers.

### 2.3 Measurement and calculation

#### 2.3.1 N_2_O concentration in soil profiles

**Figure 2:**
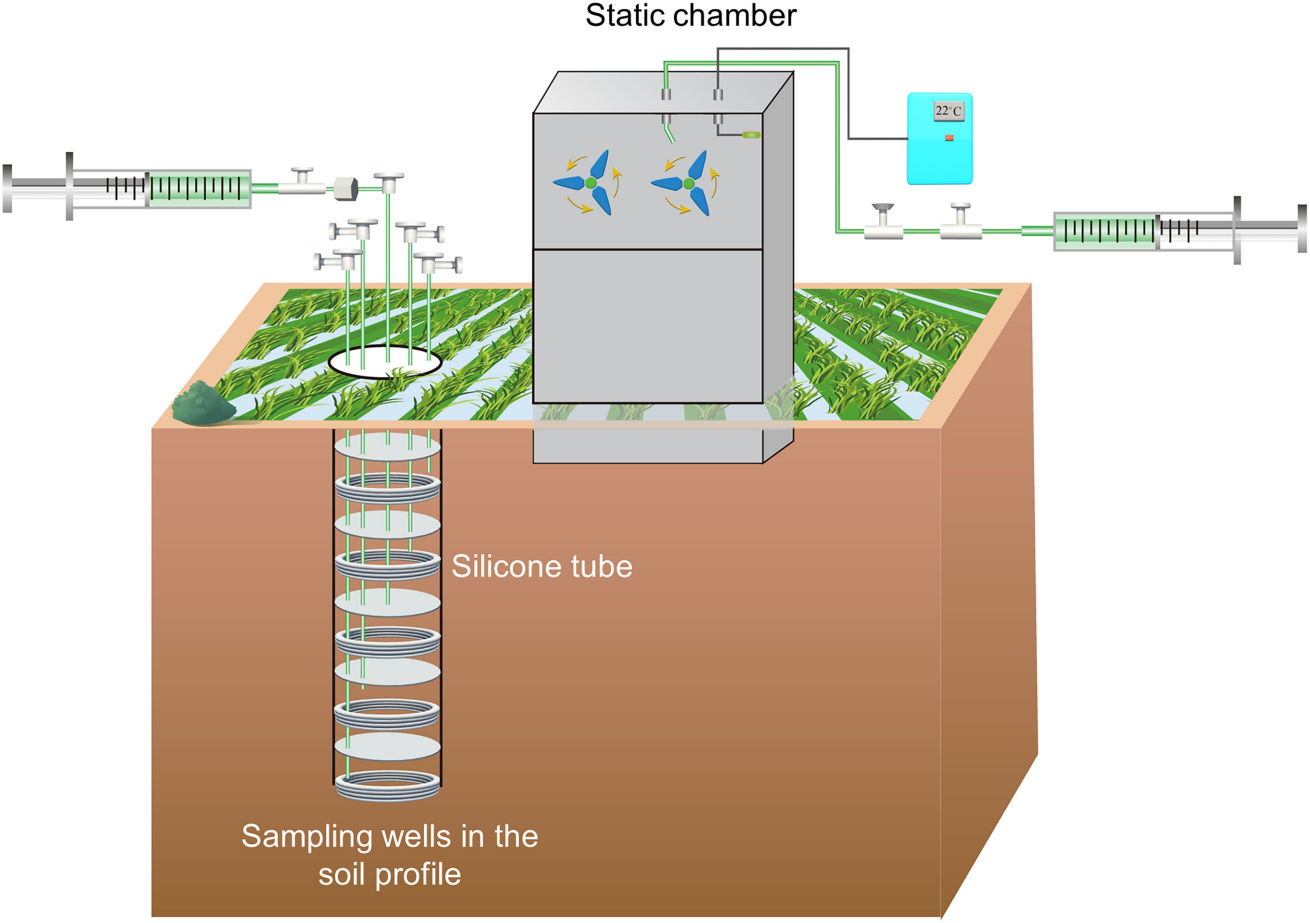
The details information about the static chamber and sampling wells in the soil profile. The static chamber consists of a fan and a digital thermometer. Sampling wells consist of five independent silicone cells and layouts at 0–50 cm depth.

N_2_O concentrations in soil profiles were collected using in-site gas multiport wells at depths of 10, 20, 30, 40, and 50 cm (**Figure 2**). Each gas sampling multiport well consisted of five individual silica-tube gas cells that allowed gas exchange but prevented liquid from entering. The gas collection events were started from seedling transplanting until harvest at an interval of 5–7 d the intervals which were adjusted to 2 d at least 3 times after fertilization. N_2_O gas samples were collected between 9:00 AM and 11:00 AM, using a 100 ml syringe connected to individual gas cells via a three-way valve, and the gas samples were then directly injected into a vacuum aluminum bag. N_2_O gas samples were measured by Agilent 7890B gas chromatograph system (Agilent Technologies, Inc., USA) to analyze N_2_O concentrations of bags.

#### 2.3.2 Soil surface N_2_O emission

Soil surface N_2_O emission was simultaneously monitored with N_2_O concentrations in soil profiles by using the static chamber method (**Figure 2**). The N_2_O sampler consisted of a standard chamber (length: 0.5 m; width: 0.5 m; height: 0.5 m), an extension chamber (length: 0.5 m; width: 0.5 m; height: 0.5 m), and a base (length: 0.5 m; width: 0.5 m; height: 0.1 m). Bases were inserted proximately 5 cm into the soil and involved 6-hill seedings after seeding transplanting. An approximate 1 cm water depth was maintained in the tanks of chambers to prevent gas exchange between the chamber and atmosphere. Gas samples were extracted using a 100 ml syringe connected to a silicone tube of the standard chamber via a three-way valve at intervals of 15 min (0, 15, and 30 min). Gas samples were immediately transported to a vacuum aluminum bag. The laboratory method for determining the concentration of gas samples coincided with N_2_O concentration in the soil profile.

The calculation method of soil surface N_2_O flux and cumulative N_2_O emission referred to our previous report (Liu et al., 2022):

(1) $$\begin{array}{c} F=\rho h\cdot dC/dt\cdot 273/(273+t)\cdot \rho /\rho_{0} \end{array}$$

where *F* represents soil surface N_2_O flux (μg m^−2^ h^−1^), ρ represents the density of N_2_O at a standard state (1.964 kg m^−3^), *h* represents the height from the soil surface to the top of the standard chamber, dC/dt represent the concentration of N_2_O varies with time (μg m^−1^ h^−1^), *t* represents the temperature of gas sample sampling, $\rho_{0}$ represents the standard atmospheric pressure (Equation 1).

(2) $$\begin{array}{c} f=\sum_{i=1}^{n}\left[\frac{F_{i}+F_{i-1}}{2}\cdot d\cdot 24\cdot 10^{-2}\right] \end{array}$$

where *f* is the cumulation N_2_O emission (kg ha^−1^), *F_i_* and *F_i+1_* are the adjacent N_2_O flux (μg m^−2^ h^−1^), *d* is the interval between *F_i_* and *F_i+1_* (Equation 2).

### 2.4 Data analysis

Prior to analysis, the Shapiro-Wilk normality test was used to examine the normal distribution characteristics of the data for each indicator. One-way analysis of variances (ANOVA) was conducted to assess whether there are significant differences in the average N_2_O concentration within soil profiles at depths of 0–10, 10–20, 20–30, 30–40, and 40–50 cm between continuously flooded irrigation (I_CF_) and alternate wetting and drying irrigation (I_AWD_) during the basal fertilizer period, tiller fertilizer period, and panicle fertilizer period in 2019 and 2020. Significant differences between treatments were subsequently identified using Tukey’s HSD test at the 5% probability level. Linear regression analysis was performed to explore the relationship between soil surface N_2_O flux and N_2_O concentrations at depths of 0–10, 10–20, 20–30, 30–40, and 40–50 cm. Partial least-squares path model (PLS-PM) was conducted to infer the direct and indirect effects of N_2_O accumulation in different soil profiles (depths of 0–10, 10–20, 20–30, 30–40, and 40–50 cm) on soil surface N_2_O emission under different irrigation regimes (I_CF_ and I_AWD_). R^2^ donates the proportion of variance explained. Values adjacent to arrows represent standardized path coefficients. The “+” “−” indicate significant positive and negative effects, and absolute value of path coefficients are proportional to the strength of the effects. The above statistical analyses were conducted and visualized using the R software (version 4.4.3) with the package of “stats” for ANOVA, the package of “lm” for linear regression analysis, and the package of “plspm” for PLS-PM.

## 3 Results

### 3.1 Vertical and temporal dynamics of soil profile N_2_O concentrations

**Figure 3:**
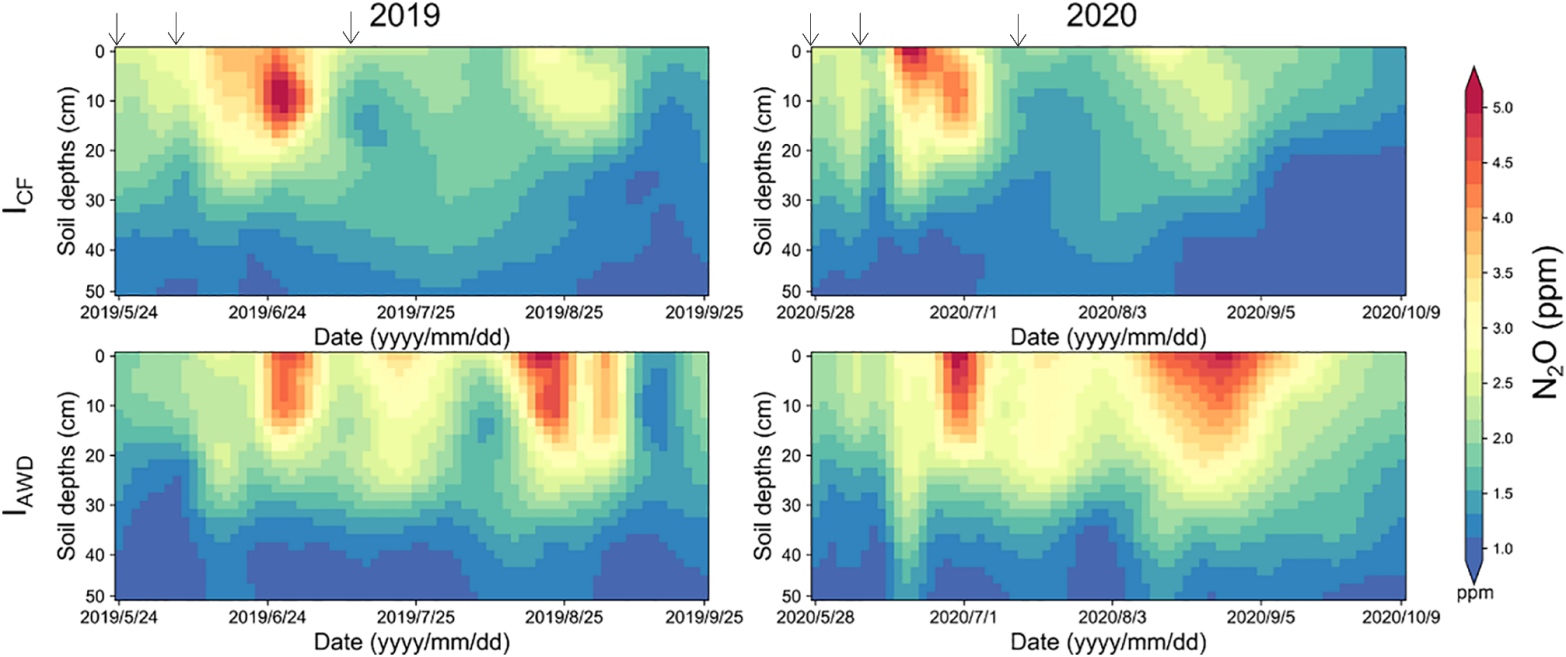
Spatial dynamics variation of N_2_O concentration in 0–50 cm soil profiles under continuously flooded irrigation (I_CF_) and alternate wetting and drying irrigation (I_AWD_) during two rice growing seasons in 2019 and 2020. The arrows indicate fertilization.

Our measurements revealed systematic patterns in how I_AWD_ irrigation reshapes the distribution and dynamics of N_2_O within the soil profile (**Figure 3**). Temporally, N_2_O concentrations peaked consistently 5–7 days after fertilization during both the tillering fertilizer period (TF) and panicle fertilizer period (PF) (**Figure 3**). In addition, the temporal dynamics of soil N_2_O was directly orchestrated by the cyclic wetting and drying phases (**Figure 3**). During TF, During TF, N_2_O peaks post-fertilization were comparable between I_AWD_ and I_CF_. The key divergence emerged during the extended mid-season drainage (water control period), resulting in significantly higher background N_2_O concentrations than in I_CF_. During PF, the synergy between nitrogen application and the intense drying cycles of I_AWD_ was striking. I_AWD_ amplified post-fertilization N_2_O peaks by 10–150% across the soil profile compared to I_CF_ (**Figure 3**).

Spatially, N_2_O concentrations exhibited a pronounced vertical gradient, decreasing progressively with soil depth (**Figure 3**). The upper 0–20 cm layer served as the dominant reservoir, maintaining concentrations of 0.71–7.90 ppm, significantly higher than the 0.35–4.47 ppm observed in the 20–50 cm layer. Crucially, I_AWD_ amplified N_2_O concentrations across the entire soil profile but with a depth-dependent magnitude (**Figure 3** and **Table 1**). The most pronounced enhancements occurred in the top 30 cm during PF, where I_AWD_ significantly increased N_2_O by 60.00–78.97% relative to I_CF_. This effect was most intense in the 0–10 cm layer (up to 78.97% increase), gradually attenuating with depth to a 14–28% increase at 40–50 cm (**Table 1**). A similar, though less pronounced, pattern was observed during TF. These results identified the upper soil profile (0–20 cm) as the epicenter of I_AWD_-induced N_2_O production and accumulation. Crucially, the enhancing effect of I_AWD_ on N_2_O concentration was not only depth-dependent but also modulated by the wetting/drying cycle intensity and duration, particularly during TF and PF.

**Table 1:** The average N_2_O concentration within soil profiles at depths of 0–10, 10–20, 20–30, 30–40, and 40–50 cm under continuously flooded irrigation (I_CF_) and alternate wetting and drying irrigation (I_AWD_) during the basal fertilizer period, tiller fertilizer period, and panicle fertilizer period in 2019 and 2020.

Periods	Soil depths (cm)	2019		2020	
		I_CF_	I_AWD_	I_CF_	I_AWD_
Basal fertilizer Period	0–10	3.04a	3.01a	3.01a	2.97a
	10–20	2.73a	2.93a	2.80a	2.88a
	20–30	2.16a	1.90a	2.21a	2.11a
	30–40	1.63a	1.54a	1.64a	1.62a
	40–50	1.29a	1.28a	1.26a	1.33a
Tiller fertilizer Period	0–10	3.47b	5.18a	3.98b	4.76a
	10–20	3.30b	4.25a	3.47b	4.17a
	20–30	2.42b	3.31a	2.37b	3.23a
	30–40	1.75a	1.91a	1.57b	2.01a
	40–50	1.34b	1.54a	1.33b	1.51a
Panicle fertilizer Period	0–10	1.88b	3.08a	2.14b	3.83a
	10–20	1.71b	2.83a	1.91b	3.09a
	20–30	1.27b	2.23a	1.50b	2.40a
	30–40	1.13b	1.50a	1.27b	1.78a
	40–50	0.92b	1.18a	1.11a	1.27a

### 3.2 Dynamics of N_2_O flux and cumulative emissions

**Figure 4:**
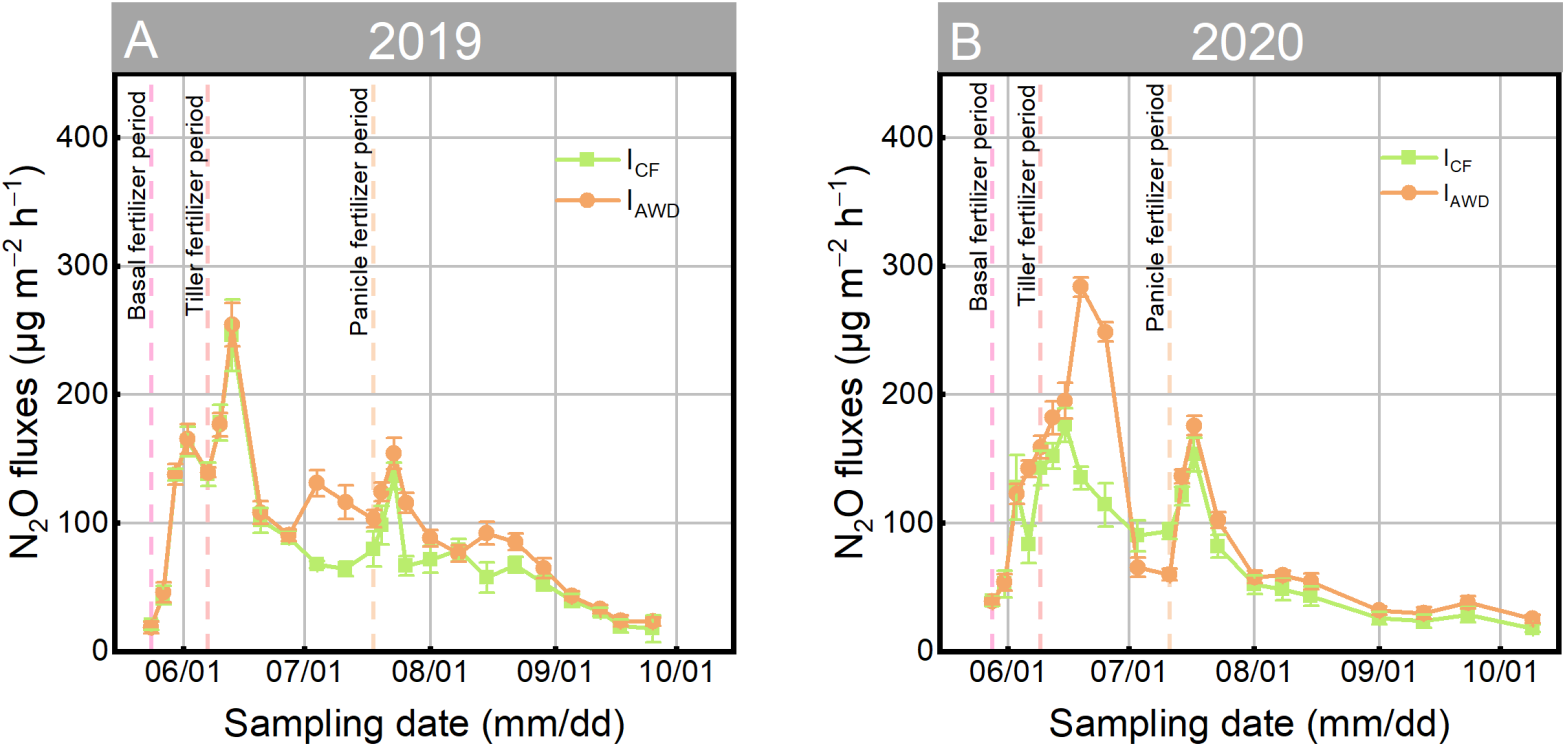
Temporal dynamics variation of N_2_O flux in soil surface under continuously flooded irrigation (I_CF_) and alternate wetting and drying irrigation (I_AWD_) during two rice-growing seasons in **(A)** 2019 and **(B)** 2020. Dash lines indicate the application of basal fertilizer, tiller fertilizer, and panicle fertilizer. Vertical bars are mean ± standard deviations (n = 3).

Surface N_2_O flux dynamics were fundamentally governed by the interplay between fertilization timing and the I_AWD_ cycles (**Figure 4**). While both irrigation regimes exhibited peak fluxes 5–7 days after fertilizer application—a period coinciding with maximum nitrogen availability—the magnitude and temporal pattern of these emissions were critically modulated by the distinct wetting and drying phases of I_AWD_ (**Figure 4**). I_AWD_ consistently generated higher peak fluxes (142.60–283.83 μg m^−2^ h^−1^) than I_CF_ (127.87–246.39 μg m^−2^ h^−1^), particularly after fertilization during the intense drying cycles of the panicle stage: a larger N_2_O reservoir had been pre-formed during the preceding dry period (**Figures 3**, **4**).

**Figure 5:**
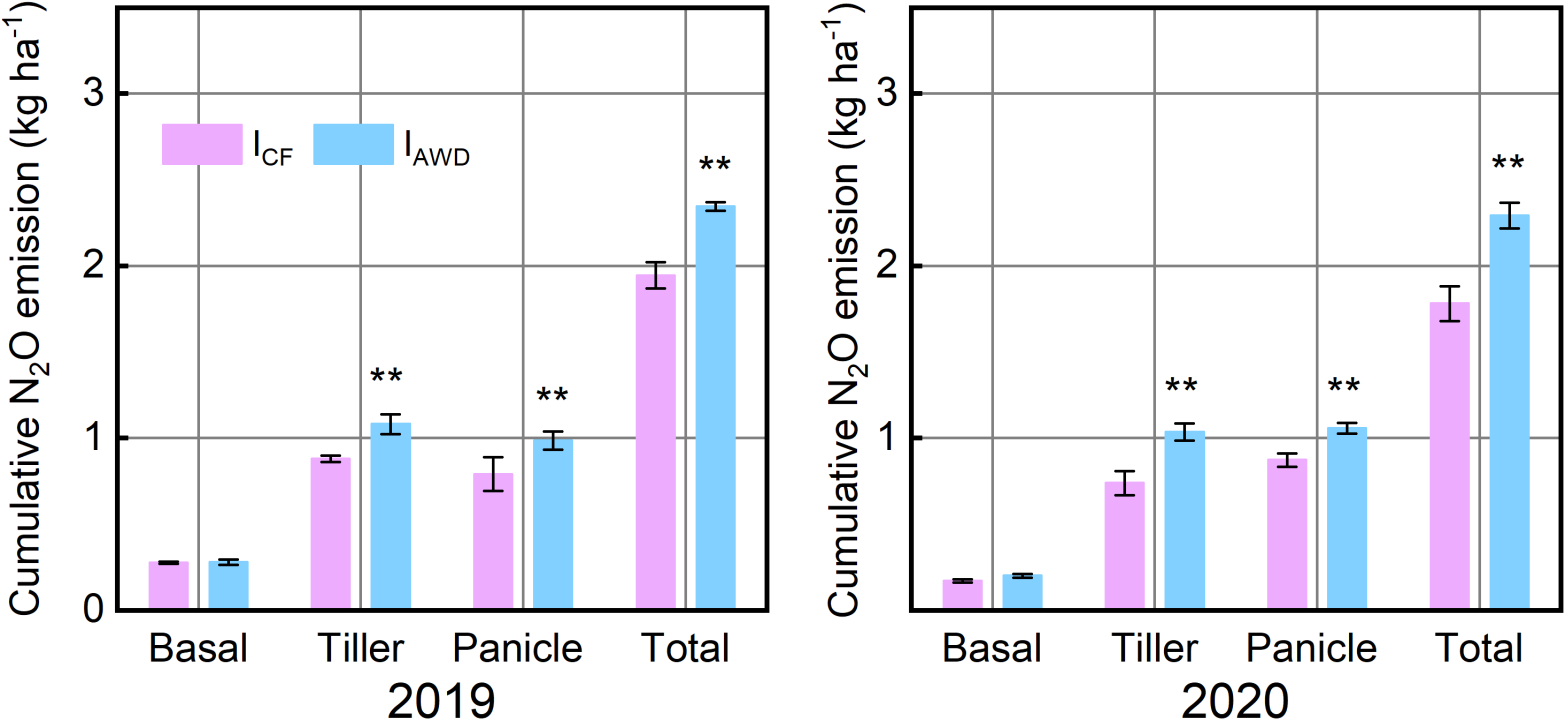
The cumulative N_2_O emission under continuously flooded irrigation (I_CF_) and alternate wetting and drying irrigation (I_AWD_) during the basalfertilizer period, tiller fertilizer period, panicle fertilizer period, and the entire rice growing season in 2019 and 2020. ** indicates significant differences at *P* < 0.01. Vertical bars are mean ± standard deviations (n=3).

This elevated flux activity under I_AWD_ translated directly into significantly increased cumulative N_2_O emissions (**Figure 5**). The effect was pronounced during both TF and PF—key windows for water and nutrient control. Across two consecutive growing seasons (2019–2020), I_AWD_ significantly enhanced cumulative N_2_O emissions by 23.02–40.13% during TF and 21.17–24.72% during PF (**Figure 5**). Consequently, the total seasonal N_2_O burden under I_AWD_ was significantly elevated by 20.59% to 28.72% compared to I_CF_ (**Figure 5**).

### 3.3 The relationship between N_2_O concentrations in soil profiles and N_2_O emissions

**Figure 6:**
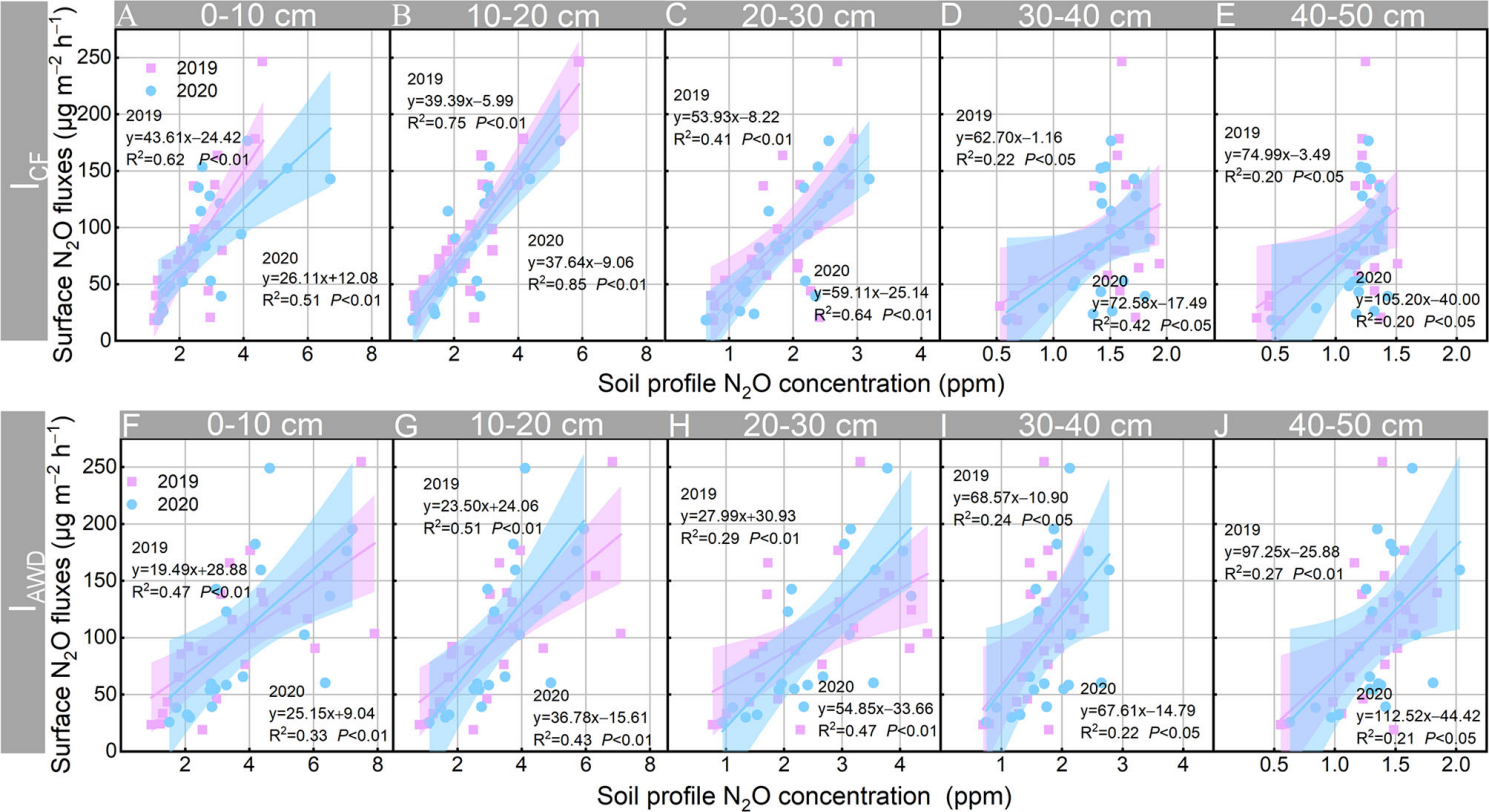
Relationship between soil surface N_2_O flux and N_2_O concentration within soil profiles at depths of 0–10, 10–20, 20–30, 30–40, and 40–50 cm under **(A–E)** continuously flooded irrigation (I_CF_) and **(F–J)** alternate wetting and drying irrigation (I_AWD_) in 2019 (n=48) and 2020 (n=42) rice growing seasons.

**Figure 7:**
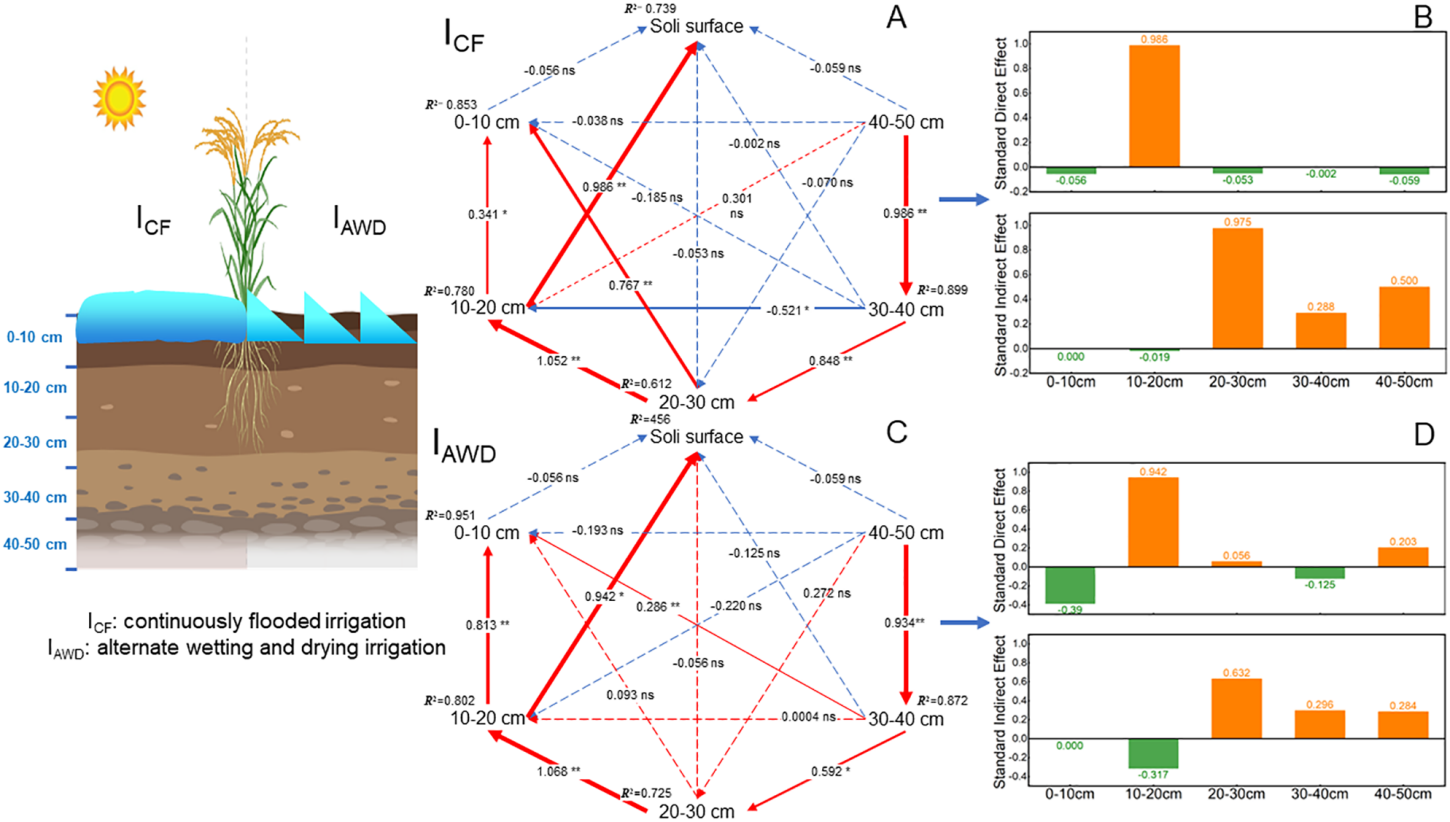
Partial least-squares path model (PLS-PM) was conducted to infer the direct and indirect effects of N_2_O accumulation in different soil profiles (depths of 0–10, 10–20, 20–30, 30–40, and 40–50 cm) on soil surface N_2_O emission under **(A, B)** I_CF_ and **(C, D)** I_AWD_. Values adjacent to arrows represent standardized path coefficients. The “+” and “−” indicate significant positive and negative effects, and absolute value of path coefficients are proportional to the strength of the effects. * and ** indicate significance at *P* < 0.05 and *P* < 0.01, respectively.

Linear regression analysis was performed to build the relationships between the N_2_O concentrations in different soil profiles and soil surface N_2_O emissions in I_CF_ and I_AWD_ paddy fields (**Figure 6**). Regardless of irrigation regimes, soil profile N_2_O concentrations significantly (*P* < 0.01 in 0–30 cm soil profile and *P* < 0.05 in 30–50 cm soil profile) positively correlated with soil surface N_2_O fluxes. Partial Least Square-Structural Equation Modelling (PLS-PM) was conducted to further quantify the contribution of soil profile N_2_O accumulation to N_2_O emissions in I_CF_ and I_AWD_ paddy fields (**Figure 7**). Soil surface N_2_O emissions are both affected by direct and indirect effects induced by N_2_O concentrations in different soil profiles. For the I_CF_ paddy fields, soil profile N_2_O concentrations totally explained 73.9% of soil surface N_2_O emissions. Soil surface N_2_O emissions were significantly (*P* < 0.01) directly affected by N_2_O concentration in the 10–20 cm soil profile (0.986) and indirectly affected by N_2_O concentration in the 20–30 cm soil profile (0.975) (**Figures 7A, B**). For the I_AWD_ paddy fields, soil profile N_2_O concentrations totally explained 45.56% of soil surface N_2_O emissions. Soil surface N_2_O emissions were significantly (*P* < 0.05) directly affected by N_2_O concentration in the 10–20 cm soil profile (0.942) and indirectly affected by N_2_O concentration in the 20–30 cm soil profile (0.632) (**Figures 7C, D**).

## 4 Discussions

### 4.1 Soil profile N_2_O concentration

Our findings robustly identify the 0–20 cm soil layer as the persistent and dominant reservoir for N_2_O accumulation in paddy fields, a phenomenon that transcends the influence of both fertilization events and irrigation regimes (**Figure 3**). This consistent vertical stratification challenges the conventional framing of N_2_O dynamics as a primarily surface-led process and establishes a generalized principle for subsurface N_2_O storage in submerged soils. The convergence of this hotspot across management practices suggests that its formation is governed by fundamental soil-biogeochemical constraints rather than water status alone (McCoy et al., 2023; Li et al., 2025). Specifically, the upper soil profile constitutes a critical zone where the abundance of fresh organic substrates from root exudates and senesced biomass, coupled with elevated microbial biomass and activity, creates a prime niche for N_2_O generation via nitrification and denitrification (Kim et al., 2022; Song et al., 2024). Furthermore, the physical-hydraulic properties of this layer, including its porosity and gas diffusivity, likely favor the entrapment and dissolved-phase storage of N_2_O following its production, rather than immediate emission (Bentzon-Tarp et al., 2023; Gao et al., 2014; Li et al., 2025). While previous studies in aerobic uplands have noted similar subsurface peaks, its confirmation in the unique, redox-fluctuating environment of paddy fields underscores a potentially universal hierarchy in soil profile gas dynamics (Li et al., 2021). A key limitation of this snapshot is its temporal scope; future investigations should employ higher-frequency monitoring to resolve how the stability of this subsurface reservoir responds to rapid redox oscillations. Moreover, integrating isotopic tracing and microbial functional gene analysis across these defined depth intervals will be crucial to definitively partition the relative contributions of nitrification versus denitrification to the observed accumulation (Qin et al., 2023; Yankelzon et al., 2024). Ultimately, explicitly acknowledging the 0–20 cm layer as a pre-eminent N_2_O reservoir redefines the target zone for mitigation, shifting the focus from managing surface emissions to controlling subsurface production and storage.

Crucially, our results demonstrate that I_AWD_ induces a depth-dependent amplification of N_2_O within the soil profile, establishing the 0–30 cm soil layer as the epicenter of this enhanced production (**Figure 3**). This finding reveals that I_AWD_’s impact is not uniform but systematically diminishes with depth, a nuance previously obscured by a primary focus on surface flux measurements. The intensified response in the top 30 cm, particularly during the panicle fertilizer stage, points to a critical interaction between management-induced redox dynamics and depth-stratified biogeochemical drivers (**Figure 3**) (Pittelkow et al., 2013; Liu et al., 2022). We posit that the frequent oxygen fluctuations under I_AWD_ preferentially stimulate nitrifier and denitrifier activity in the upper horizons, where the confluence of labile carbon from root exudates and fresh fertilizer nitrogen is greatest (Chen et al., 2014; Tang et al., 2025). Furthermore, the physical-hydraulic properties of the soil matrix create a “bio-geochemical filter”: while the saturated conditions during flooding phases may limit gas diffusion and trap N_2_O in solution (Islam et al., 2022), the subsequent drying phases enhance gaseous release from upper layers, thereby “resetting” the system for another cycle of production upon rewetting (Verhoeven et al., 2018; Grohs et al., 2024). This cyclic entrapment and pulsed production mechanism is inherently more pronounced in the biologically active topsoil than in the consistently anoxic subsoil, explaining the observed depth gradient. While previous studies have documented the overall stimulatory effect of I_AWD_ on N_2_O, our profile data move beyond this established fact to delineate the precise vertical zonation of its impact (Chen et al., 2022; Liu et al., 2022; Sha et al., 2022). A limitation of this mechanistic interpretation is the challenge in disentangling the *in-situ* production from the potential vertical transport of N_2_O. Future research employing isotopic techniques or *in-situ* sensors for O_2_ and N_2_O at a finer spatial-temporal resolution is crucial to partition the contribution of localized production versus physical transport in shaping this depth-dependent response. Ultimately, confirming that I_AWD_’s primary influence is confined to the agriculturally manageable plough layer offers a clear target for developing stratified mitigation strategies, such as deep fertilizer placement or the use of subsoiling to physically disrupt the dominant production zone.

### 4.2 The relationship between soil profile N_2_O concentration and emission

Our partial least-squares path model (PLS-PM) pinpoints the 10–20 cm soil layer as the dominant biogeochemical hotspot, exerting the strongest direct control on surface N_2_O fluxes in I_AWD_ paddy fields (**Figure 7**). This finding moves beyond simply identifying where N_2_O accumulates and reveals which specific subsurface zone mechanistically governs its atmospheric release. While previous research has established strong correlations between surface emissions and N_2_O concentrations in the upper soil profile (e.g., 0–15 cm) (Li et al., 2021), our path analysis provides causal-weight evidence that the 10–20 cm layer is the pivotal engine room (**Figure 7**). We posit that this specific depth represents a critical interface where optimal conditions for N_2_O production converge with efficient gas transport pathways. During I_AWD_ drying phases, the 10–20 cm layer transitions from anoxic to sub-oxic states, creating a thermodynamically favorable niche for both nitrification and denitrification, fueled by the diffusion of ammonium from deeper fertilizer bands and labile carbon from the rhizosphere (Kravchenko et al., 2017; Marushchak et al., 2021). Crucially, unlike the often-saturated and diffusion-limited layers below 20 cm, this horizon remains sufficiently connected to the atmosphere during drainage, allowing the produced N_2_O to escape rather than be further reduced to N_2_ (Wang et al., 2023). This creates a “Goldilocks Zone” for N_2_O emission—deep enough to accumulate substantial substrate from the plough layer but shallow enough to facilitate efficient egress (Kravchenko et al., 2017). The stronger explanatory power of our model under I_CF_ compared to I_AWD_ suggests that the stability of the saturated environment creates a more predictable, diffusion-dominated system. In contrast, the hydraulic perturbations of I_AWD_ introduce greater variability in gas transport pathways, albeit with the 10–20 cm layer remaining the unequivocal primary controller (Liu et al., 2022). A key limitation is that our study resolves the “what” and “where” but not the precise microbial kinetics at this interface. Future research integrating *in-situ*, depth-resolved metatranscriptomics with gas diffusivity measurements is essential to unravel the relative contributions of nitrifier denitrification versus fungal denitrification to the observed dominance of this layer. Confirming the 10–20 cm layer as the command center for emissions provides a powerful new target for mitigation, suggesting that strategies like deep-point placement of enhanced-efficiency fertilizers or the sub-surface application of biochar could directly disrupt this critical nexus, potentially decoupling water savings from climate impact.

The consistent identification of the 0–20 cm layer as the primary N_2_O reservoir and the 10–20 cm layer as the dominant source of N_2_O emissions in our paddy system is consistent with findings from diverse agroecosystems outside Asia, indicating a widespread principle governed by soil-gas dynamics rather than specific pedoclimatic conditions. In temperate grasslands (Barneze et al., 2024), the lack of a direct correlation between subsurface N_2_O concentrations and surface fluxes underscores the critical, and often overlooked, role of *in-situ* consumption during gaseous diffusion—a process that can decouple production from emission. This aligns with the classical concepts of N_2_O entrapment, dissolution, and reduction during transport through the soil matrix, as detailed by Clough et al. (2005). Crucially, our observation that I_AWD_ most strongly amplifies N_2_O in the top 30 cm, coupled with the PLS-PM identification of the 10–20 cm layer as the cardinal controller of surface flux, provides a clear spatial target for these processes. It suggests that under I_AWD_, the “active layer” for N_2_O production is shallow, but the “effective layer” governing its ultimate release is slightly deeper, where production, temporary entrapment, and partial reduction interact. This dynamic is analogous to mechanisms observed in cold climates, where spring-thaw N_2_O bursts are driven more by rapid production in surface layers than by the release of deeply trapped gases (Wagner-Riddle et al., 2008). Similarly, in an Italian rice paddy system, Verhoeven et al. (2018) also found that the N_2_O emissions were associated with enhanced nitrification in the upper soil layers during plant establishment. Therefore, our lysimeter-based evidence from Asian paddy fields demonstrates that the hierarchy of subsurface N_2_O processes—wherein specific depth zones disproportionately control emissions—could be a transferable concept across contrasting environments. However, considering the current paucity of well-established relationships between soil profile gas concentrations and surface emissions on a global scale, future research ought to be carried out under a broader spectrum of climatic, soil, and crop conditions.

## 5 Conclusions

In conclusion, our study shifts the paradigm for understanding N_2_O emissions from I_AWD_ paddy systems by establishing the soil profile—not just the surface—as the critical domain. We demonstrate that the widely documented emission increase under I_AWD_ is fundamentally underpinned by a depth-dependent amplification of N_2_O within the soil matrix, with the upper 0–20 cm layer identified as the consistent and dominant production reservoir. Crucially, the application of PLS-PM transcends correlation to reveal causation, pinpointing the 10–20 cm depth as the cardinal hotspot that exerts the strongest direct control on surface fluxes. By quantitatively mapping the subsurface architecture of N_2_O production and its functional linkage to emissions, our work provides an actionable blueprint for mitigation. It compellingly argues that the 10–20 cm soil layer should be the primary target for intervention, guiding the development of straightforward and targeted strategies—such as deep-point placement of enhanced-efficiency fertilizers or subsurface biochar application—designed to directly disrupt this key emission nexus and foster a more sustainable balance between water conservation and climate security in global rice production.

## Data Availability

The raw data supporting the conclusions of this article will be made available by the authors, without undue reservation.
